# Supplementary material for: Association of Antiphospholipid Antibodies with Clinical Manifestations in Children with Systemic Lupus Erythematosus
Source: J Clin Med. 2023 Feb 10;12(4):1424. doi: 10.3390/jcm12041424 (PMC9967991; doi:10.3390/jcm12041424)
Supplement: Supplementary file 1 [file jcm-12-01424-s001.zip › jcm-2193402-supplementary.pdf]

Table S1 (ex "Table, according to the reviewer's suggestion")

Table: values of antiphospholipid antibodies of aPLA + patients

Patient No	LA			aCLA IgM			aCLA IgG			β2GPI IgM			β2GPI IgG		
Sex (F/M)	beg	1.y	3.y	beg	1.y	3y	beg	1.y	3.y	beg	1.y	3.y	beg	1.y	3.y
1 F	1.08	1	2.1	25.9	3.2	0.1	15.4	4.1	0.7	5.3	38	6.2	12	9.8	2.8
2 F	0.98	1	0.9	3.1	0.6	2.8	4.2	0.1	3.5	-	-	-	-	-	-
3 F	1.1	0.8	1.4	14.6	12	8.1	9.7	4.9	7.4	31	36	-	2.4	4.6	-
4 M	0.81	0.9	0.8	33.5	7.9	2.1	57.5	11.8	14	6.1	4.3	-	5.8	9.2	-
5 F	5.69	1.7	1.38	18	7.4	16.3	9.8	16.8	10	8.3	14.6	6.2	3.4	5.4	4.8
6 F	0.9	0.9	1.7	51	48	51.9	5.9	6.2	3.3	11.2	12	11.8	8.5	6.9	5.6
7 F	1.11	0.9	0.9	4.7	200	22.5	8.6	4.6	15.8	6.3	200	14.3	7.2	2.9	6.3
8 F	1.32	2.04	1.3	5.5	6.4	-	32.8	16	-	33.2	26	26.7	21.2	19	6.3
9 F	1.47	1.65	1.4	24	23.1	25.6	13.5	16	14.2	9.1	11.2	5.4	24.4	26	28.3
10 F	2.6	2.1	1.9	20.4	13	14.3	29.6	18	19.2	9.8	6.8	8	16.3	12.3	4
11 M	1.1	1.5	1.6	23.5	18.5	6.5	7.5	6.2	4.8	-	-	-	-	-	-
12 F	1.8	2.34	2.8	120	62	24	110.9	51	21.4	38.4	9.2	5	19.2	4.3	6
13 M	0.9	1.8	2.1	20.7	13.7	14	50.7	25.6	28	19.6	12	14.2	4.7	5.4	7.8
14 M	0.96	1.1	0.8	10.7	12.6	11	17.5	16.8	18	3.5	4	3.6	6.1	4.2	5.6
15 M	2.3	3.1	0.6	-	-	-	-	-	-	-	-	-	-	-	-
16 F	0.8	1	0.9	17.1	8.4	12.6	12.6	10.8	18	6.4	18	19.2	4.2	5.1	4.4
17 M	1.1	0.8	1	6.2	12.2	18.4	16.2	22.2	22	18.2	19.2	18	21.8	16.2	25

beg - at the beginning, 1.y after one year of follow-up, 3.y after three years of follow-up

aCLA, β2GP (IgM, IgG) - PLU/ml, - not measured
